# Sex-dependent effects of prenatal food and protein restriction on offspring physiology in rats and mice: systematic review and meta-analyses

**DOI:** 10.1186/s13293-021-00365-4

**Published:** 2021-02-09

**Authors:** Julian K. Christians, Haroop K. Shergill, Arianne Y. K. Albert

**Affiliations:** 1 Department of Biological Sciences, Simon Fraser University, 8888 University Drive, Burnaby, British Columbia V5A 1S6 Canada; 2 Centre for Cell Biology, Development and Disease, Simon Fraser University, Burnaby, Canada; 3 British Columbia Children’s Hospital Research Institute, Vancouver, BC Canada; 4 Women’s Health Research Institute, BC Women’s Hospital and Health Centre, Vancouver, British Columbia Canada

**Keywords:** Developmental origins, Developmental programming, Maternal nutrition, Malnutrition, Prenatal exposure

## Abstract

**Background:**

Males and females may experience different effects of early-life adversity on life-long health. One hypothesis is that male foetuses invest more in foetal growth and relatively less in placental growth, and that this makes them susceptible to poor nutrition in utero, particularly if nutrition is reduced part-way through gestation.

**Objectives:**

Our objectives were to examine whether (1) food and/ or protein restriction in rats and mice has consistent sex-dependent effects, (2) sex-dependency differs between types of outcomes, and (3) males are more severely affected when restriction starts part-way through gestation.

**Data sources:**

PubMed and Web of Science were searched to identify eligible studies.

**Study eligibility criteria:**

Eligible studies described controlled experiments that restricted protein or food during gestation in rats or mice, examined physiological traits in offspring from manipulated pregnancies, and tested whether effects differed between males and females.

**Results:**

Our search identified 292 articles, of which the full texts of 72 were assessed, and 65 were included for further synthesis. A majority (50) used Wistar or Sprague-Dawley rats and so these were the primary focus. Among studies in which maternal diet was restricted for the duration of gestation, no type of trait was consistently more severely affected in one particular sex, although blood pressure was generally increased in both sexes. Meta-analysis found no difference between sexes in the effect of protein restriction throughout gestation on blood pressure. Among studies restricting food in the latter half of gestation only, there were again few consistent sex-dependent effects, although three studies found blood pressure was increased in males only. Meta-analysis found that food restriction in the second half of gestation increased adult blood pressure in both sexes, with a significantly greater effect in males. Birthweight was consistently reduced in both sexes, a result confirmed by meta-analysis.

**Conclusions:**

We found little support for the hypotheses that males are more affected by food and protein restriction, or that effects are particularly severe if nutrition is reduced part-way through gestation. However, less than half of the studies tested for sex by maternal diet interactions to identify sex-dependent effects. As a result, many reported sex-specific effects may be false positives.

**Supplementary Information:**

The online version contains supplementary material available at 10.1186/s13293-021-00365-4.

## Background

Males and females may experience different effects of early-life adversity on long-term health outcomes including cognition and mental health [1–5], as well as metabolic, cardiovascular, and renal disease [6–8]. It has been suggested that male foetuses may be more vulnerable to food shortage [9], and others have since suggested that males may have greater susceptibility to early-life adversity more generally [4]. This difference may be due to a male strategy to prioritize growth, even in the face of adversity, whereas females are more conservative in allocating resources to growth and show more physiological adjustments to challenges [1, 2]. A potential explanation for this difference in early-life strategies is that there is greater competition for mates among males, making it adaptive to prioritize growth and risk early death, rather than prioritize short-term survival and risk later life reproductive opportunities. Elevated competition among males is one theory to explain sexual size dimorphism in mammals [10, 11], and indeed early postnatal growth has been linked to later life reproductive strategy in male humans [12]. A related hypothesis is that, to maximize foetal growth, males invest less in placental growth, and as a result have to make compensatory investments in the placenta at the expense of the foetus if they encounter poor nutrition later in pregnancy [9].

These hypotheses are particularly relevant to prenatal insults such as malnutrition, but are difficult to test in humans. Many studies rely on associations with birthweight, but this is multifactorial and not determined solely by maternal diet. Studies of famines are informative, and some are able to examine effects of malnutrition at specific time points in gestation [13], but these are confounded by other aspects of maternal stress. Carefully controlled experiments with animal models are therefore needed to understand sex-dependent responses to early-life adversity. Rodents are frequently used as models to investigate the effects of prenatal maternal nutrition on offspring health [14–16] and, given sexual dimorphism and competition among males, rodents would be expected to show similar sex-dependent strategies to humans.

The purpose of this systematic review is to examine the effects of food and protein restriction in rats and mice to test the hypothesis that impaired prenatal nutrition has greater effects on males than on females. We predict that, if male foetuses invest more in foetal growth and relatively less in placental growth [9], and are less responsive to stress signals [1, 2], then food restriction beginning mid-gestation will have particularly deleterious effects on males. Exposure to nutritional restriction throughout pregnancy is expected to constrain the growth of both sexes, whereas if food quantity and/or quality decreases abruptly part-way through gestation, males will be disproportionately affected if they have prioritized growth and maintained less reserve capacity earlier in pregnancy [9]. We sought to assess whether (1) food and/ or protein restriction in rats and mice has consistent sex-dependent effects, (2) sex-dependency differs between types of outcomes, and (3) males are more severely affected when restriction starts part-way through gestation.

## Methods

We followed the Preferred Reporting Items for Systematic Reviews and Meta-Analyses (PRISMA) guidelines [17]. The review protocol is described below and has not been registered.

### Data sources and search

PubMed and Web of Science were searched using the terms: (prenatal OR maternal) AND (fetal OR fetus) AND (rat OR rodent OR mouse OR mice) AND (sex specific OR sex dependent OR sex difference OR gender difference OR gender specific) AND (restriction OR restricted OR undernutrition OR undernourished OR malnutrition OR “low protein” OR deprivation) on June 4, 2020.

### Eligibility criteria

The participants, interventions, comparisons, outcomes, and study design (PICOS) criteria were as follows: participants were mice or rats; intervention was macronutrient restriction (protein or food) during gestation (manipulations may have been introduced prior to pregnancy or may have been conducted for only part of pregnancy); comparison was with a control diet provided ad libitum; outcomes were examined in offspring from manipulated pregnancies (i.e. effects on subsequent generations were not included); study design was a controlled experiment where offspring sex was taken into account (either by analysing the sexes separately, or by testing for a statistical interaction between treatment and sex). Only studies published in English were included. Reviews were excluded.

### Study selection, data items, and summary measures

Titles and abstracts were screened for relevance. Eligible full texts were assessed to extract the following variables: species and strain, manipulation (protein vs. food restriction), timing and duration of manipulation, severity of the manipulation, sample size per group (i.e. the number of dams manipulated), effects of manipulation on litter size and sex ratio, how offspring sex was taken into account (i.e. analysing the sexes separately or testing for an interaction), and how/whether analyses accounted for multiple offspring per dam. In addition, we recorded offspring traits that were reported to be affected by the manipulation in one or both sexes. Traits were not included where sexes were pooled.

Where other treatments in addition to protein or food restriction were involved, we focused on the effects of protein or food restriction on controls, e.g. if offspring were weaned onto a high-fat diet or control diet, we only included the results from offspring on the control diet. Similarly, where cross-fostering of pups allowed the effects of manipulation during pregnancy to be distinguished from those of manipulation during lactation, we included only the effects of prenatal manipulation.

Because we focused on animal studies, for which protocols are generally not published ahead of time, we could not assess outcome reporting bias. We assumed that animals were assigned to experimental groups at random, and therefore that there was little risk of bias within individual studies. We acknowledge that statistically significant effects were more likely to be reported.

### Meta-analyses

We performed meta-analyses on traits measured in three or more studies with similar protocols where the trait was measured at the same age. Where data were provided in figures, they were extracted using WebPlotDigitizer [18].We used random effects meta-analysis as implemented in the R package ‘metafor’ [19] to calculate the estimated average standardized mean difference (SMD). SMD were weighted by the inverse variance, which gives greater weight to larger studies. The I^2^ metric was calculated to assess study heterogeneity. We conducted a moderated meta-analysis to compare the effects in males vs. the effects in females. This involves estimating the treatment effect in males and females separately in each study, and then including sex as a moderator in the meta-regression. We then estimated whether the treatment effect differed by sex. Residual variance was allowed to differ in each sex within each study.

## Results

### Study selection

**Fig. 1:**
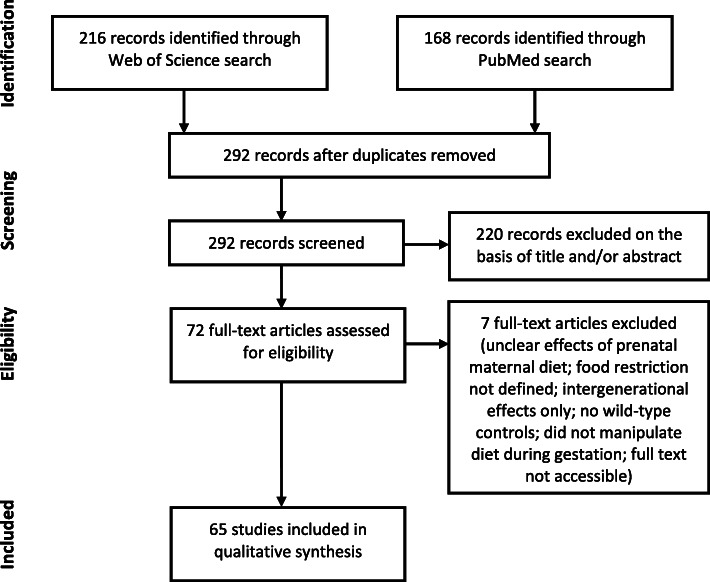
PRISMA flow diagram showing selection process (figure template from [17])

The PubMed search identified 168 results, while the Web of Science search identified 216 and 292 remained after deduplication (Fig. 1). After screening titles and abstracts for relevance, the full texts of 72 articles were assessed, with a further 7 excluded at this stage (2 did not clearly distinguish effects of prenatal maternal diet from other effects; 1 did not define the level of food restriction; 1 only reported effects on a subsequent generation; 1 did not include wild-type control animals; 1 manipulated diet prior to mating but not during gestation; 1 full text from 1994 could not be accessed), and 65 included for further synthesis.

### Study characteristics

All data extracted from the 65 studies are provided in Additional file 1. Of these studies, 29 used Wistar rats (23 performed protein restriction [20–42] and 6 performed food restriction [43–48]), 21 used Sprague-Dawley rats (7 protein restriction [49–55] and 14 food restriction [56–69]), 3 used other strains of rats (2 protein restriction with Wistar-Kyoto [70, 71] and 1 food restriction with Long Evans [72]) and 12 used various strains of mice (7 protein restriction [73–79] and 5 food restriction [80–84]). We therefore focused primarily on studies of Wistar and Sprague-Dawley rats.

### Sex-dependent effects of food or protein restriction in rats

We categorized offspring traits (Table 1) to facilitate comparisons of different types of outcomes. To address the question of whether food and/ or protein restriction has consistent sex-dependent effects, we then summarized the types of traits that were affected to a greater extent in males, those affected to a greater extent in females, and those affected in both sexes.

**Table 1:** Categories used to group traits

Category^**a**^	Examples of traits
Adrenal	gene expression (mRNA or protein); organ weight
Behavioural	stereotypic responses; food intake; food preference
Blood lipids	cholesterol, LDL, NEFA, triglycerides
Bone	bone mineral content, bone mineral density
Brain	gene expression (mRNA or protein)
Cardiovascular	blood pressure; measures of vascular or ventricular function; heart rate; gene expression (mRNA or protein) in heart or vasculature; organ weight
Fat	body fat percentage, fat pad weight
Hepatic	hepatic gene expression, enzyme activity, glycogen, blood proteins produced by liver; organ weight
Lung	alveolar number, gene expression (mRNA or protein); triolein uptake; organ weight
Muscular	fibre density, diameter
Oxidative status	plasma carbonyl, glutathione, thiols and melatonin, superoxide anion scavenging activity, oxidative status score based on plasma biomarkers
Pancreatic	beta-cell mass; islet size; gene expression (mRNA or protein); organ weight
Placental	gene expression (mRNA or protein), SOD activity
Renal	gene expression (mRNA or protein); urine production; ion excretion; glomerular number; glomerular filtration rate; organ weight
Spleen	organ weight

^a^Body weight, blood hormones, and blood glucose were not categorized

We first focused on studies in which maternal diet was restricted for the duration of gestation in Wistar or Sprague-Dawley rats (Table 2). To reduce heterogeneity in experimental design, this summary excluded 4 studies of Wistar rats [23, 25, 36, 37] and 1 of Sprague-Dawley rats [50] that also manipulated diet throughout lactation (without cross-fostering to distinguish effects of gestation and lactation), and 1 study of Sprague-Dawley rats that began restriction 3 weeks before breeding [49]. Although excluded from Table 2, the results of these studies are summarized in Additional file 1.

**Table 2:** Traits affected by food restriction (FR) or protein restriction (PR) throughout gestation in rats

Study	***N***^a^	Manipulation	Traits affected in males	Traits affected in females	Traits affected in both sexes	Analysed sexes separately or tested interaction
**Wistar rats**						
Howie 2012 [43]	6	FR (50%)	Insulin:leptin ratio; blood lipids; hepatic; spleen	Hepatic	Birthweight; body weight; fat; bone; leptin; blood lipids	Sexes separately
Ozaki 2001 [44]	10–11	FR (70%)	Cardiovascular	Cardiovascular	Cardiovascular	Sexes separately
Peiris 2010 [45]	?	FR (30%)	Insulin	Fat	Body weight	Interaction
Sánchez-Garrido 2013 [46]	?	FR (70%)	Body weight; insulin^d^	Body weight; insulin^d^	Birthweight; FSH	Sexes separately
Alwasel 2009 [20]	?	PR (9% vs 18%)	None	None	Cardiovascular; renal	Interaction
Bellinger 2006^b^ [22]	8–11	PR (9% vs 18%)	Body weight; fat	Fat; behavioural	None	Interaction
Cooke 2014^c^ [24]	6	PR (8% vs 20%)	Renal	Renal	Foetal weight; renal	Sexes separately
Elmes 2007 [26]	8	PR (9% vs 18%)	Cardiovascular	None	Cardiovascular	Interaction
Elmes 2009 [27]	6	PR (9% vs 18%)	Cardiovascular^e^	Cardiovascular^e^	Cardiovascular	Interaction
Kwong 2006^b^ [28]	11–13	PR (9% vs 18%)	None	None	None	Not clear
Kwong 2007^b^ [29]	11–13	PR (9% vs 18%)	Hepatic	None	None	Sexes separately
Langley-Evans 1997 [30]	3	PR (9% vs 18%)	Lung; renal; brain	None	Body weight; cardiovascular	Sexes separately
Langley-Evans 2005 [31]	7	PR (9% vs 18%)	Hepatic	None	None	Interaction
Langley-Evans 2006 [32]	5–8	PR (9% vs 18%)	None	Placental weight; hepatic	Placental weight	Interaction
Mallinson 2007^b^ [33]	11–13	PR (9% vs 18%)	Muscular	None	None	Interaction
McMullen 2005 [34]	6	PR (9% vs 18%)	None	Cardiovascular; renal	Cardiovascular	Interaction
McMullen 2005 [35]	10–11	PR (9% vs 18%)	None	Renal	Renal	Interaction
Theys 2009 [38]	7	PR (8% vs 20%)	Blood lipids; pancreatic	Pancreatic; hepatic	Birthweight; pancreatic	Interaction
Torrens 2009 [39]	6–7	PR (9% vs 18%)	Cardiovascular; blood inflammatory markers	None	Cardiovascular	Sexes separately
Vega 2016 [40]	6	PR (10% vs 20%)	Placental; foetal hepatic^e^; blood lipids; hepatic	Placental; foetal hepatic^e^; fat; hepatic	Foetal weight; foetal:placental ratio; placental; foetal hepatic; hepatic; leptin; corticosterone; insulin	Sexes separately
Ye 2018 [41]	7–10	PR (10% vs 20%)	None	Brain; behavioural	None	Interaction
Zambrano 2006 [42]	4–6	PR (10% vs 20%)	Leptin; glucose; blood lipids	Birthweight; fat	Insulin	Sexes separately
**Sprague-Dawley rats**						
Gao 2012^d^ [51]	10	PR (6% vs 20%)	Placental	Placental	Foetal weight; placental weight; placental	Interaction
Gao 2012^d^ [52]	10	PR (6% vs 20%)	Placental	Placental	Foetal weight; placental	Interaction
Sathishkumar 2012 [53]	8–9	PR (6% vs 20%)	Cardiovascular	Cardiovascular	Cardiovascular	Sexes separately
Woods 2004^b^ [54]	6–9	PR (5% vs 19%)	Body weight; renal	None	Body weight; cardiovascular; renal	Sexes separately and interaction
Woods 2005 [55]	13–16	PR (9% vs 19%)	Renal	None	Birthweight	Sexes separately

^a^Sample size is provided as the number of dams per group (? = not stated)

^b^Study also manipulated diet during various periods of gestation. This table includes only results of manipulation throughout gestation

^c^Study also includes a group where manipulation continued through lactation. This table includes only results from dams collected during gestation

^d^Study collected at day 14 and 18 of gestation

^e^Manipulation had opposite effect in males and females for some traits

In both Wistar and Sprague-Dawley rats (Table 2), no type of trait was consistently more severely affected in one particular sex. For example, cardiovascular traits were studied most frequently, and some studies found such traits to be more affected in males, while others found females to be more affected, and other studies found some traits to be more affected in males and some to be more affected in females. Considering all types of traits, more studies (8 out of 27) found some traits to be more affected in males and no traits to be more affected in females, compared with the reverse scenario (4 out of 27). However, of the 12 studies that found sex-dependent effects in one sex but not the other, the proportion with male-specific effects was not significantly greater than half (*p* = 0.39, two-tailed binomial test).

Some traits were examined in more than one study, allowing more detailed assessment of the consistency of sex-dependent effects. Considering offspring of Wistar and Sprague-Dawley dams protein-restricted for the duration of gestation (studies listed in Table 2; complete data provided in Additional file 1), birthweight was reduced in both sexes in one study [38], but in another was reduced in females but not males [42]. Conversely, weight at 3 weeks [54] and 7 weeks of age [30] was reduced in both sexes, whereas weight at 4 [22], 22, and 35 weeks [54] was reduced only in males. Blood pressure was generally increased in both sexes [20, 26, 30, 34, 54], although in one study, this was true at 6 months of age, but at 3 months, only males were affected [53]. Heart rate was sometimes increased in both sexes [26], sometimes in females only [34]. Blood triglycerides were increased in males in two studies [38, 42]. Serum insulin was increased in both sexes [40, 42], as was serum leptin in one study [40], although leptin was increased only in males in another [42].

**Fig. 2:**
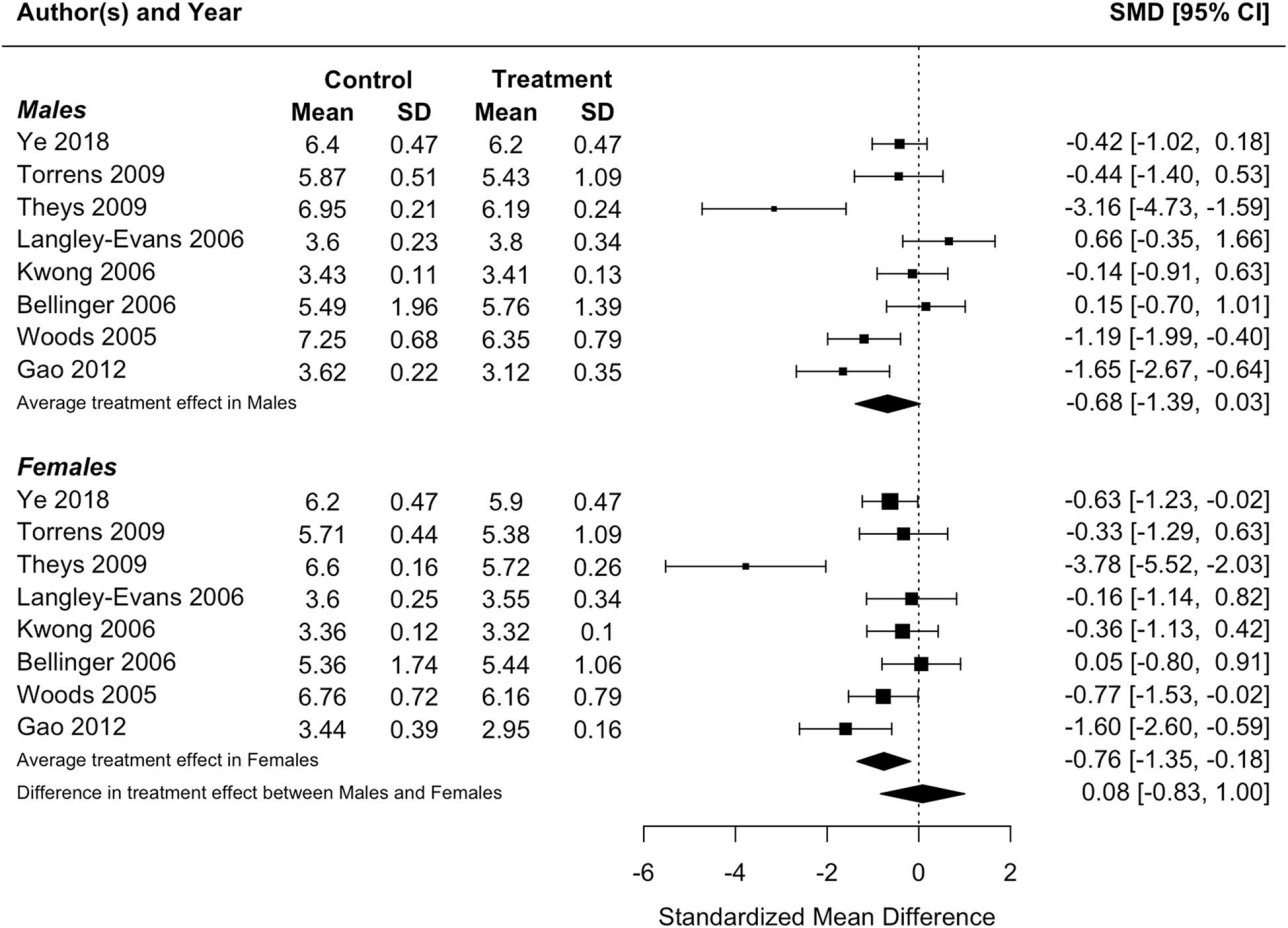
Meta-analysis of the effects of protein restriction throughout gestation on body weight around birth in rats (foetal day 20 (Kwong 2006 [28]) (Langley-Evans 2006 [32]); foetal day 21 (Gao 2012 [51]); postnatal day 0 (Bellinger 2006 [22]) (Theys 2009 [38])(Torrens 2009 [39]) (Ye 2018 [41]); postnatal day 1 (Woods 2005 [55]))

**Fig. 3:**
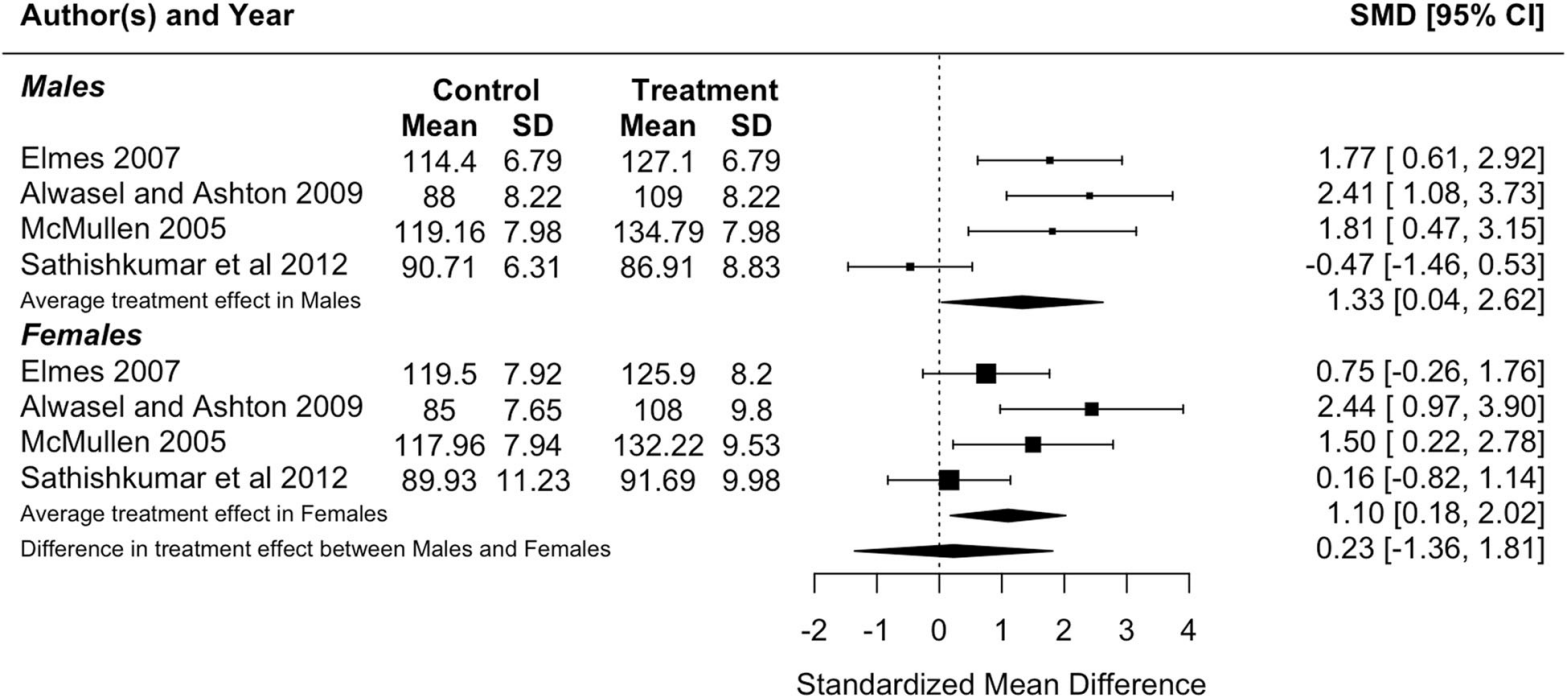
Meta-analysis of the effects of protein restriction throughout gestation on blood pressure in juvenile rats (mean blood pressure at 4 weeks (Alwasel and Ashton 2009 [20]); systolic blood pressure at 4 weeks (McMullen 2005 [34]) (Elmes 2007 [26]); mean blood pressure at 1 month (Sathishkumar et al 2012 [53]))

**Fig. 4:**
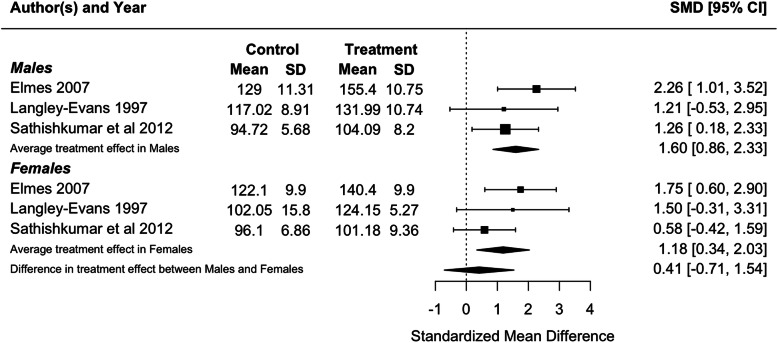
Meta-analysis of the effects of protein restriction throughout gestation on blood pressure in adolescent rats (systolic blood pressure at 7 weeks (Langley-Evans 1997 [30]); systolic blood pressure at 8 weeks (Elmes 2007 [26]); mean blood pressure at 3 months (Sathishkumar et al 2012 [53]))

Because blood pressure and birthweight (including weight at foetal day 20 or 21 or postnatal day 1) were measured in three or more studies, we performed meta-analyses for these traits. Protein restriction for the duration of gestation reduced birthweight in females (*p* = 0.01), and tended to reduce birthweight in males, although the latter difference was marginally non-significant (*p* = 0.06; Fig. 2). There was no difference in the effect of protein restriction between males and females (*p* = 0.86). Protein restriction for the duration of gestation increased blood pressure in juvenile (4–5 weeks old; Fig. 3) and adolescent (7–13 weeks old; Fig. 4) males (juveniles: *p* = 0.04; adolescents: *p* < 0.0001) and females (juveniles: *p* = 0.02; adolescents: *p* = 0.006), but there was no difference in effect between males and females (juveniles: *p* = 0.78; adolescents: *p* = 0.47). The data extracted from individual studies for the meta-analyses are provided in Additional file 1.

### Effects of food or protein restriction occurring in the latter half of gestation

To assess our prediction that males would be more severely affected when restriction starts part-way through gestation, we examined studies manipulating maternal diet for part of gestation. Fourteen studies applied food restriction in the latter half of gestation (generally beginning at gestational day (GD) 10 or 11) in Sprague-Dawley rats (Table 3). Of these 14 studies, 4 found some traits to be more affected in males and no traits to be more affected in females, whereas no study found the reverse (Table 3; complete data provided in Additional file 1). However, 4 out of 4 studies finding sex-dependent effects in males but not females is not significantly greater than half, i.e. if studies finding female-specific effects but no male-specific effects were equally likely (*p* = 0.13, two-tailed binomial test).

**Table 3:** Traits affected by food restriction (FR) in the latter half of gestation in Sprague-Dawley rats

Study	***N***^a^	Manipulation	Duration	Traits affected in males	Traits affected in females	Traits affected in both sexes	Analysed sexes separately or tested interaction
Choi 2007 [56]	6	FR (75%)	GD 10–birth	Hepatic	Hepatic	Birthweight; hepatic	Sexes separately
Desai 2005 [57]	12	FR (50%)	GD 10–birth	None	None	Body weight	Not clear
Desai 2007 [58]	6	FR (50%)	GD 10–birth	None	None	Body weight; fat; leptin; blood lipids; glucose; insulin; glucose tolerance test; behavioural	Not clear
Gutierrez-Arzapalo 2018 [59]	3–5	FR (50%)	GD 11–birth	Cardiovascular^b^	Cardiovascular^b^	Birthweight; cardiovascular	Interaction
Hemmings 2005 [60]	8–11	FR (40%)	GD 15–birth	Cardiovascular	Cardiovascular	Birthweight	Sexes separately
Khorram 2011 [61]	4–6	FR (50%)	GD 10–birth	Corticosterone; adrenal^a^	Adrenal^a^	Body weight; adrenal	Sexes separately
Lee 2013 [62]	4	FR (50%)	GD 10–birth	Body weight	None	Body weight; fat; blood lipids; leptin	Sexes separately
Matveyenko 2010 [63]	6	FR (50%)	GD 11–21	Body weight; pancreatic	None	Birthweight; pancreatic	Sexes separately
Molle 2015 [64]	6	FR (50%)	GD 10–birth	Behavioural^b^; brain	Behavioural^b^	Behavioural; brain	Interaction
Munoz-Valverde 2015 [65]	12	FR (50%)	GD 11–birth	None	None	Birthweight; cardiovascular; hepatic; fat; glucose	Sexes separately
Paek 2015 [66]	4	FR (50%)	GD 10–birth	None	None	Birthweight; body weight; lung	Not clear
Rodríguez-Rodríguez 2015 [67]	6	FR (50%)	GD 11–birth	Cardiovascular; oxidative status	None	Birthweight; oxidative status	Sexes separately
Rodríguez-Rodríguez 2017 [68]	5	FR (50%)	GD 11–birth	Cardiovascular^b^	Cardiovascular^b^	Birthweight; cardiovascular	Interaction
You 2015 [69]	5	FR (50%)	GD 10–birth	Hepatic; homocysteine	None	None	Sexes separately

^a^Sample size is provided as the number of dams per group (? = not stated)

^b^Manipulation had opposite effect in males and females for some traits

Birthweight (including weight 1 day after birth) was consistently reduced in both sexes [56, 59, 60, 63, 65–68]. Similarly, body weight was reduced in both sexes at 7 days [65] and at 21 days [66], but was increased in both sexes at 6 months and 9 months of age [58, 61, 62], although some studies found it to be reduced in males only at 3 days [62] and 21 days [63]. Blood pressure was increased in males only [59, 67, 68].

**Fig. 5:**
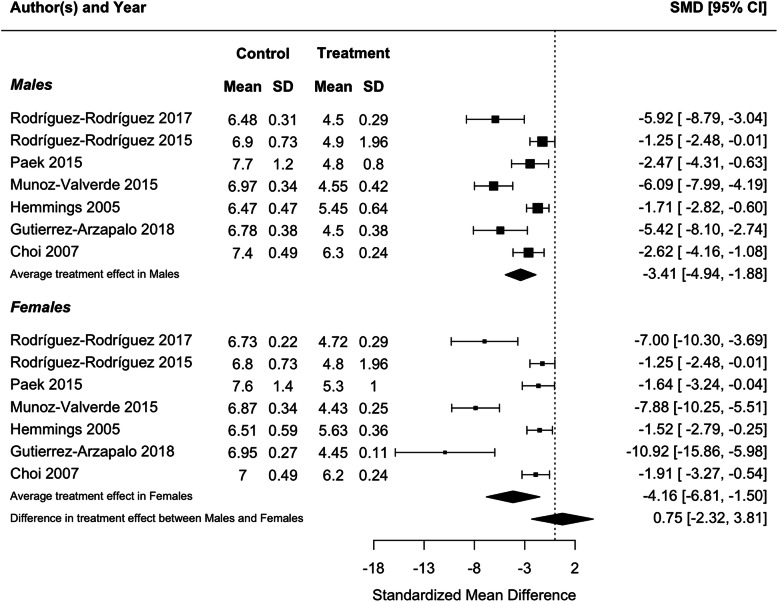
Meta-analysis of the effects of food restriction in the second half of gestation on body weight around birth in rats (postnatal day 0 (Hemmings 2005 [60]); postnatal day 1 (Choi 2007 [56]) (Gutierrez-Arzapalo 2018 [59]) (Munoz-Valverde 2015 [65]) (Paek 2015 [66]) (Rodriguez-Rodriguez 2015 [67]) (Rodriguez-Rodriguez 2017 [68]))

**Fig. 6:**
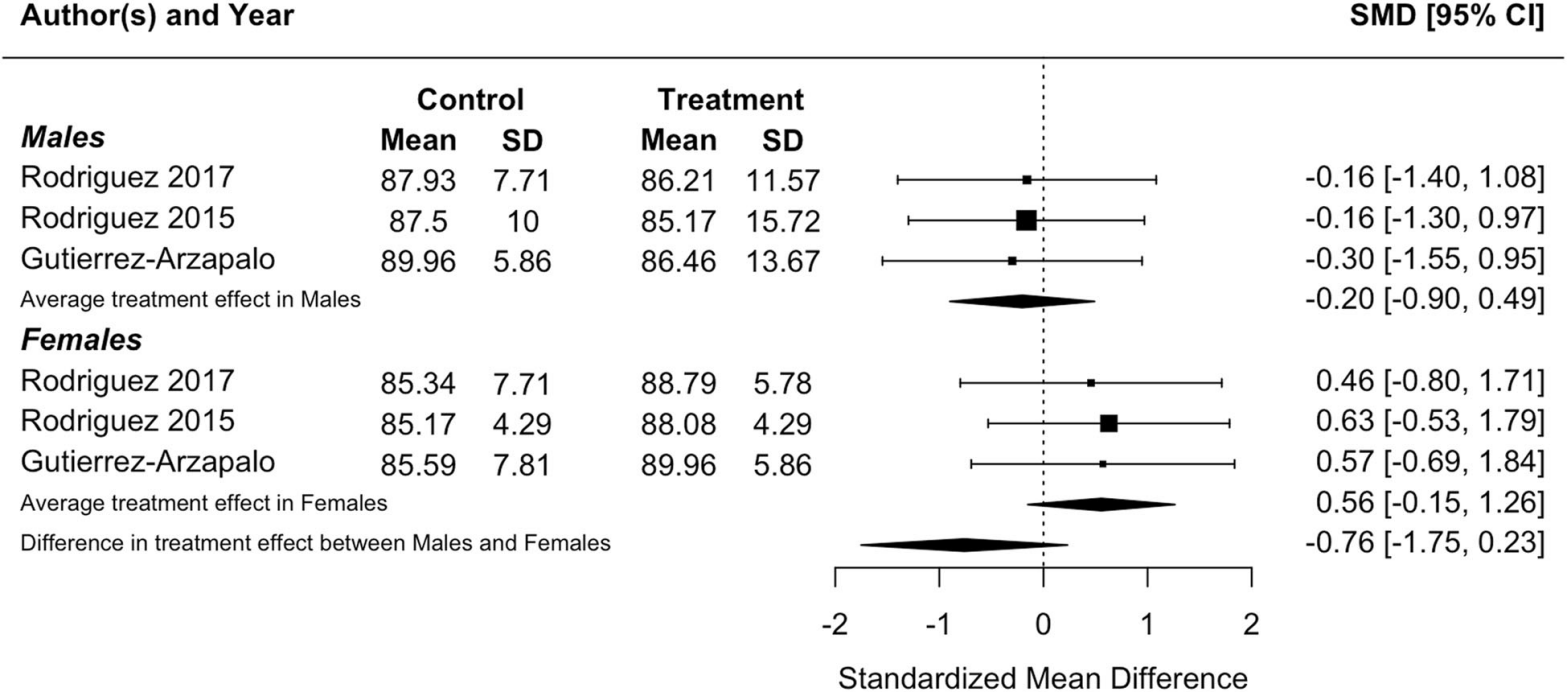
Meta-analysis of the effects of food restriction in the second half of gestation on systolic blood pressure in juvenile (21 day old) rats (Gutierrez-Arzapalo 2018 [59]) (Rodriguez-Rodriguez 2015 [67])(Rodriguez-Rodriguez 2017 [68])

**Fig. 7:**
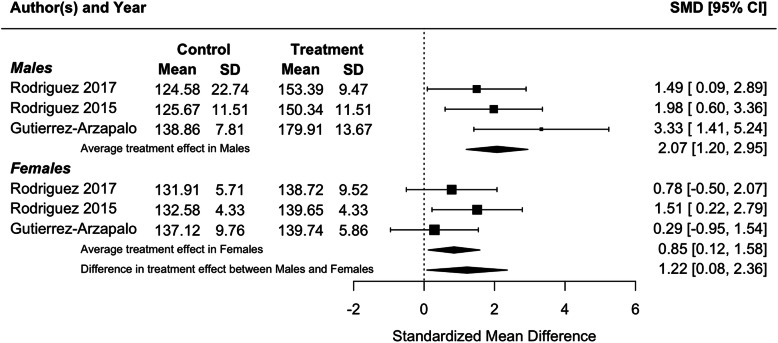
Meta-analysis of the effects of food restriction in the second half of gestation on systolic blood pressure in adult (6 month old) rats (Gutierrez-Arzapalo 2018 [59]) (Rodriguez-Rodriguez 2015 [67]) (Rodriguez-Rodriguez 2017 [68])

Consistent with the results of individual studies, a meta-analysis found that food restriction in the second half of gestation reduced birthweight in females (*p* = 0.002) and males (*p* < 0.0001), but that there was no difference in the effect of protein restriction between males and females (Fig. 5; *p* = 0.63). Meta-analysis of food restriction in the second half of gestation found no effect on blood pressure in juveniles (4–5 weeks old; Fig. 6), and no difference in effect between males and females (*p* = 0.13). However, in adults (6 months old), a meta-analysis found that blood pressure was increased in both males (*p* < 0.0001) and females (*p* = 0.02), with a significantly greater effect in males (*p* = 0.04; Fig. 7), in contrast to results from individual studies, where effects were significant in males only [59, 67, 68]. The data extracted from individual studies for the meta-analyses are provided in Additional file 1.

Five studies used multiple groups to manipulate protein levels throughout different periods of gestation (4 in Wistar rats and 1 in Sprague-Dawley rats), allowing effects of restriction late in gestation to be compared with restriction beginning earlier (Table 4). Maternal protein restriction from GD 0–7 or from GD 15–22 but not from GD 8–14 increased hepatic glycogen concentration in male offspring only [21]. Male-specific effects on 4-week weight and 9-month gonadal fat were similar whether protein restriction occurred from GD 15–22 or throughout gestation, although restriction in the last week of gestation also decreased 2-month weight in males only [22]. While some measures of pancreatic islets at day 1 and pancreatic expression of some genes at day 21 were affected in males only by restriction in the last week of pregnancy, no such male-specific effects were observed in offspring of dams restricted throughout gestation and lactation [23]. In contrast, 4-week density of slow fibres in the gastrocnemius muscle was increased in males only by restriction throughout gestation, whereas there were no male-specific effects when restriction occurred in the last week of gestation [33]. Protein restriction, whether from GD 11 to birth or from the beginning of gestation to birth, reduced body weight and glomerular volume at 22 weeks in males but not females, although the reduction in glomerular volume was significantly greater in offspring who experienced protein restriction throughout gestation [54].

**Table 4:** Traits affected by protein restriction (PR) among studies that manipulated diet in multiple periods of gestation

Study	***N***^a^	Manipulation	Duration	Traits affectedin males	Traits affectedin females	Traits affectedin both sexes	Analysed sexes separatelyor tested interaction
**Wistar rats**							
Bellinger 2005 [21]	5	PR(9% vs 18%)	GD 0–7	Hepatic	Behavioural	Body weight	Interaction
			GD 8–14	None	Behavioural	None	
			GD 15–22	Hepatic	Behavioural	Body weight	
Bellinger2006 [22]	8–11	PR(9% vs 18%)	GD 0–birth	Body weight; fat	Fat; behavioural	None	Interaction
			GD 0–7	Body weight;fat; behavioural	Body weight; fat	None	
			GD 8–14	Body weight;behavioural	None	None	
			GD 15–22	Body weight;fat	Fat; behavioural	None	
Chamson-Reig2006 [23]	3–4	PR(8% vs 20%)	GD 0–weaning	None	None	Pancreatic	Interaction
			GD 0–14	None	Pancreatic	Pancreatic	
			GD 8–14	None	Pancreatic	Pancreatic	
			GD 15–22	Pancreatic	Pancreatic	Pancreatic	
Mallinson2007 [33]	11–13	PR(9% vs 18%)	GD 0–birth	Muscular	None	None	Interaction
			GD 0–7	Muscular	None	Muscular	
			GD 8–14	None	Muscular	Muscular	
			GD 15–22	None	None	Muscular	
**Sprague-Dawley rats**							
Woods2004 [54]	6–9	PR(5% vs 19%)	GD 1–birth	Body weight;renal	None	Body weight; cardiovascular; renal	Sexes separately andinteraction
			GD 1–11	None	None	None	
			GD 11–birth	Body weight;renal	None	Cardiovascular	

^a^Sample size is provided as the number of dams per group (? = not stated)

### Sex-dependent effects of food or protein restriction in mice

Only 12 studies of mice met our inclusion criteria, and of these, experimental protocols were more variable than those of the rat studies, with a greater proportion beginning maternal nutrient restriction before mating, or continuing restriction through at least part of lactation. Overall, there was no clear pattern of males being more affected than females (Additional file 1). Two studies of C57BL/6J mice had protocols most comparable with those of the rat studies described above (protein restriction throughout gestation only). In one case, blood pressure at 6 months was increased in male offspring of dams subjected to nutrient restriction, but was decreased in female offspring [73], whereas blood pressure at 20 weeks was increased in both sexes in another study [74].

### Analytical approaches

The number of dams per treatment ranged from 3 to 18, with a median of 7; a few studies did not report sample sizes in terms of the number of dams (Additional file 1). While most studies found some sex-specific effects, those that did not include studies with both small (3) and moderate (~ 12) sample sizes (Additional file 1), suggesting that the detection of sex-specific effects was not entirely a function of statistical power. In studies where the prenatal environment is manipulated, it is the dam, not the individual offspring, that is the unit of replication. Therefore, it is necessary to account for the use of multiple pups per litter to avoid pseudoreplication. In most studies, this was achieved by using only one or two animals of each sex from each litter for a given measurement. Less than half of the studies (28/65) reported testing for the sex by maternal diet interaction to identify sex-dependent effects. Even in studies that stated that interactions were used, the p-values for the interaction for specific tests were often not reported.

## Discussion

A number of authors have suggested that human males may have greater susceptibility to early-life effects because of a strategy to prioritize growth [1, 2, 4, 9]. The purpose of the present study was to test whether male rats and mice are more susceptible to the effects of food and protein restriction during gestation, experimental approaches that are frequently used to model the development of health and disease in humans. We first sought to assess whether food and/ or protein restriction in rodents has consistent sex-dependent effects, and whether sex-dependency differs between types of outcomes, and found few consistent patterns. Where the same trait was studied in more than one study in offspring of rat dams protein-restricted for the duration of gestation, sex-specific effects were generally not consistent, although blood pressure was generally increased in both sexes. Whether diet was manipulated throughout gestation or in the latter half of pregnancy, a greater number of studies found some traits affected in males and no traits affected in females rather than the reverse pattern. However, the proportion of studies finding effects in males but not females was not significantly greater than half, and a large majority of studies found at least some traits more severely affected in females.

Given the hypothesis that male foetuses invest more in foetal growth and relatively less in placental growth [9], we predicted that nutritional restriction beginning mid-gestation will have particularly deleterious effects on males. There was some support for this prediction: When food or protein was restricted throughout gestation, blood pressure was generally affected in both sexes, whereas in three studies where food was restricted in the latter half of pregnancy, blood pressure was affected in males only. However, in a single study that used separate experimental groups to examine protein restriction throughout gestation and in the latter half of gestation only, blood pressure was increased in both sexes with both windows of exposure [54]. More generally, where studies used multiple groups to manipulate protein levels throughout different periods of gestation, there were not consistently more effects when restriction occurred in the latter half of gestation.

The literature we reviewed focused on effects in surviving offspring, but if males are more susceptible to nutritional restriction during gestation, we predict that males would be more likely to die before birth. However, of the 65 studies we examined, 23 found no effect on litter size and only 2 reported a reduction in litter size in nutrient restricted dams; the remainder of the studies did not report effects on litter size (Additional file 1). Although these results suggest that maternal nutrient restriction did not increase male mortality, only 5 studies investigated sex ratio at birth, with none reporting an effect of maternal diet. Nevertheless, in future studies, it would be useful to report sex ratio in case the sexes differ in their susceptibility to the manipulation.

A potential contributor to the lack of clear patterns is that this area of research often suffers from a lack of robust statistical testing, whereby authors investigate sex-dependence by testing the sexes separately, rather than explicitly testing the statistical interaction between sex and early-life environment. In this review, less than half of the studies described testing interactions, which has been reported previously for studies of humans [1, 4] and of other animal models [85]. The problem with testing the sexes separately can be understood by considering a scenario where an early-life insult has similar effects in males and females. If a study has statistical power of 0.8 to detect such an effect (which is generally considered adequate power, and higher than in many studies [86]), but analyses the sexes separately, then the probability of the effect being significant in both sexes is 0.8 × 0.8 = 0.64, while the probability of being significant in neither sex is 0.2 × 0.2 = 0.04, and the probability of being significant in one sex but not the other is 0.32. Therefore, even though the effect is similar in both sexes, there is a substantial probability that it will be reported as “sex-specific” if the sexes are analysed separately. As a result, many reports of sex-specific effects may in fact be false positives. Conversely, testing the sexes separately will miss some “sex-dependent” effects, i.e. those that are present in both sexes, but differ in magnitude. For example, our meta-analysis found that food restriction in the second half of gestation significantly increased adult blood pressure in both males and females, but that this effect was significantly greater in males. Tests of statistical interactions (e.g. between sex and early-life environment) have lower power than tests of main effects (e.g. effect of sex or effect of early-life environment). However, the solution to this issue is not to accept inadequate statistical approaches, but rather to increase sample sizes to provide appropriate power for robust testing.

Protein and food restriction in rodents is widely used to model prenatal malnutrition in pregnancy, which has clear effects on adult health in humans [13]. Contrary to the hypothesis that males are more sensitive to food shortage, women but not men exposed to famine in early gestation had higher adult mortality risk [87] and higher adult BMI [88]. In a separate cohort, famine exposure elevated adult blood pressure and increased the risk of hypertension in women only [89]. However, exposure to famine early in gestation affected brain size in males but not females [90], and exposure in the second trimester increased the risk of affective disorders in males only [91].

## Perspectives and significance

A number of studies have suggested that males have greater susceptibility to early-life adversity, with some hypothesizing that reduced investment in placental growth makes males particularly vulnerable to poor nutrition. This hypothesis makes clear predictions that (1) males will be more affected by food and protein restriction, and (2) effects will be particularly severe if nutrition is reduced part-way through gestation. We reviewed rat and mouse studies of food and protein restriction to test these predictions and found little support for either. Future work should examine potential sex-dependent effects with robust statistical approaches (e.g. interaction terms) to avoid both false positives (i.e. effects that are erroneously reported as sex-specific) and false negatives (i.e. effects that are not recognized as sex-dependent because they are significant in both sexes, even though they differ in magnitude).

## Supplementary Information

**Additional file 1:** Data extracted from 65 studies meeting eligibility criteria, including more detailed data extracted for meta-analyses.

## Data Availability

This review was based on published data.

## Abbreviations

BMI: Body mass index
FR: Food restriction
GD: Gestational day
PR: Protein restriction
